# New PRPS1 variant p.(Met68Leu) located in the dimerization area identified in a French CMTX5 patient

**DOI:** 10.1002/mgg3.875

**Published:** 2019-07-23

**Authors:** Justine Lerat, Corinne Magdelaine, Paco Derouault, Hélène Beauvais‐Dzugan, Eric Bieth, Blandine Acket, Marie‐Christine Arne‐Bes, Franck Sturtz, Anne‐Sophie Lia

**Affiliations:** ^1^ Univ. Limoges, MMNP Limoges France; ^2^ CHU Limoges, Service Oto‐Rhino‐Laryngologie et Chirurgie Cervico‐Faciale Limoges France; ^3^ CHU Limoges, Service Biochimie et Génétique Moléculaire Limoges France; ^4^ CHU Toulouse, Service Génétique Médicale Toulouse France; ^5^ CHU Toulouse, Explorations neurophysiologiques, Centre SLA, Centre de référence de pathologie neuromusculaire Toulouse France

**Keywords:** Charcot‐marie‐tooth, deafness, neuropathy, NGS, PRPS1

## Abstract

**Background:**

CMTX5 is characterized by peripheral neuropathy, early‐onset sensorineural hearing impairment, and optic neuropathy. Only seven variants have been reported and no genotype‐phenotype correlations have yet been established. PRPS1 has a crystallographic structure, as it is composed of three dimers that constitute a hexamer.

**Methods:**

Next‐generation sequencing (NGS) was performed using a custom 92‐gene panel designed for the diagnosis of Charcot‐Marie‐Tooth (CMT) and associated neuropathies.

**Results:**

We report the case of a 35‐year‐old male, who had presented CMT and hearing loss since childhood associated to bilateral optic neuropathy without any sign of retinitis pigmentosa. A new hemizygous variant on chromosomic position X:106,882,604, in the *PRPS1* gene, c.202A > T, p.(Met68Leu) was found. This change is predicted to lead to an altered affinity between the different subunits in the dimer, thereby may prevent the hexamer formation.

**Conclusion:**

CMTX5 is probably under‐diagnosed, as an overlap among the different features due to *PRPS1* exists. Patients who developed polyneuropathy associated to sensorineural deafness and optic atrophy during childhood should be assessed for *PRPS1.*

## 1 INTRODUCTION

Phosphoribosyl pyrophosphate synthetase (PRS) I deficiency is a rare medical condition caused by missense variants in *PRPS1* that lead to four different phenotypes: Arts Syndrome (MIM 301835), X‐linked Charcot‐Marie‐Tooth (CMTX5, MIM 311070), X‐linked non‐syndromic sensorineural deafness (DFNX1, MIM 304500), and Phosphoribosylpyrophosphate Synthetase Superactivity (MIM 300661). All four are X‐linked recessively inherited and in two of them, the males affected display variable degrees of central and peripheral nervous system involvement. Hearing loss is the only feature common to all four disorders and the only symptom observed in DFNX1 or DFN2.

CMTX5 is characterized by peripheral neuropathy, early‐onset sensorineural hearing impairment, and optic neuropathy. Hypotonia, gait disturbances, and loss of deep tendon reflexes with an onset between 10 and 12 years of age have also been reported (de Brouwer, 2010; Kim, 2007). Only seven variants have been reported and no genotype‐phenotype correlations have yet been established (Kim, 2007; Nishikura, 2018; Park, 2013; Robusto, 2015; Synofzik, 2014). PRPS1 has a crystallographic structure, as it is composed of three dimers that constitute a hexamer (Li, 2007). We report the case of a 35‐year‐old male, who had presented CMT and deafness since childhood associated to a new variant in *PRPS1*. Phenotype‐genotype correlation of these specific features have been looked for thanks to a review of the literature.

## 2 MATERIALS AND METHODS

### 2.1 Patient

The patient was a 35‐year‐old male who originally presented features of CMT at the age of 8 years. Peripheral blood was collected in EDTA tubes after giving informed consent. The protocol was in accordance with French ethics legislation.

### 2.2 Pathogenic variant detection

Using the reference sequence NM_002764.3 for *PRPS1* gene.

Genomic DNA was extracted using standard methods (Illustra DNA Extraction kit BACC3, GEHC). Next‐generation sequencing (NGS) was performed using a custom 92‐gene panel designed for the diagnosis of CMT and associated neuropathies (Table S1). It included the 44 known CMT genes, 27 genes involved in hereditary sensitive neuropathy (HSN) and hereditary motor neuropathy (HMN) and 21 genes associated with other neuropathies. The amplified library was prepared with the Ion P1 HiQ Template OT2 200 kit (Ampliseq Custom, Life Technologies), sequenced on a Proton sequencer (Life Technologies), and mapped to the human reference sequence GHCh38. Variants were evaluated with Alamut Mutation Interpretation Software (Interactive Biosoftware, Rouen, France) using the NM_002764.3 reference sequence for the *PRPS1* gene. Databases, such as ExAC Genome browser (http://exac.broadinstitute.org), dbSNP135 (National Center for Biotechnology Information [NCBI], Bethesda, Maryland, USA, http://www.ncbi.nlm.nih.gov/projects/SNP/), Clin Var (www.ncbi.nlm.nih.gov/clinvar), and HGMD professional (www.hgmd.cf.ac.uk), were also screened. Pathogenic variants of interest were verified by Sanger sequencing using forward and reverse primer pairs. The *PRPS1* variant was submitted to the corresponding LOVD database at http://databases.lovd.nl/shared/variants/0000438212 (patient ID00207340).

MLPA and Sanger sequencing of *GJB2* and *GJB6* were performed to screen for hearing loss.

### 2.3 3D Protein model

The tridimensional structure of the Ribose‐phosphate pyrophosphokinase 1 protein was obtained from the crystal structure of the human PRS‐I protein (Protein Database, PDB:2H06; https://www.rcsb.org) (Li, 2007).

### 2.4 Review of the literature

A review of the literature was performed, based on PubMed (https://www.ncbi.nlm.nih.gov/pubmed) and all published articles reporting pathogenic variants of *PRPS1* and neuropathies were collected. Seven variants have already been reported for *PRPS1* and CMTX5.

## 3 RESULTS

### 3.1 Clinical description

The patient was a 35‐year‐old male, who had suffered from sensory and motor neuropathy since the age of eight. The symptoms were more important and severe in the lower limbs. Amyotrophy was present in limb extremities, especially in the lower limbs. No arched feet were observed. Step walking is observed and the patient cannot walk anymore. Electrophysiological studies revealed a mixed neuropathy, with a median Nerve Conduction Velocity (NCV) of 20–30 m/s on electromyogram, associated to polyphasic responses and an amplitude of 400 µV. At the age of 28, there was an absence of sensory activity in the four limbs. The motor activity was slumped down in the four limbs, all below 11 mV, associated with very slow down NCV in the lower limbs, in a more moderate way in the upper limbs. At the age of 30, results were similar for sensory activity, motor amplitudes were even lower with very slow NCV. Sural nerve biopsy did not show any demyelinating characteristic, in childhood. No mental retardation was observed. Profound bilateral sensorineural hearing loss had been diagnosed before one year of age. Auditory Brainstem Responses (ABR) could not be carried out. CT‐scan and MRI of the petrous bones were normal. There was no tinnitus but gait disturbances were present. Hearing aids were inefficient and were removed at the age of 15. Communication was based on sign language, but with some difficulties due to reduced hand mobility. Bilateral optic neuropathy without any sign of retinitis pigmentosa was also present. He was a sporadic case and came from a non‐consanguineous family.

### 3.2 Genetic testing

**Figure 1:**
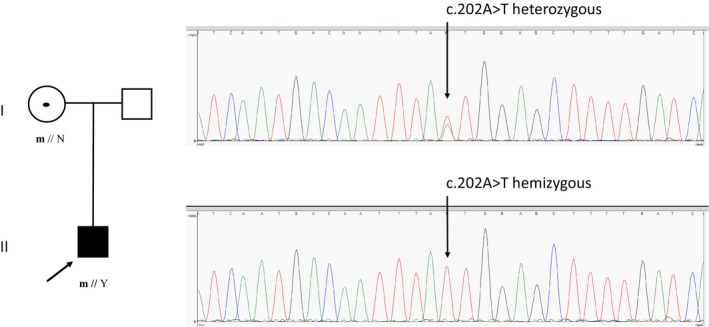
Pedigree and Sanger sequence chromatogram showing the detection of the hemizygous variant in the patient on chromosomic position X:106,882,604, c.202A>T, p.(Met68Leu) and the heterozygous variant in his unaffected mother

**Figure 2:**
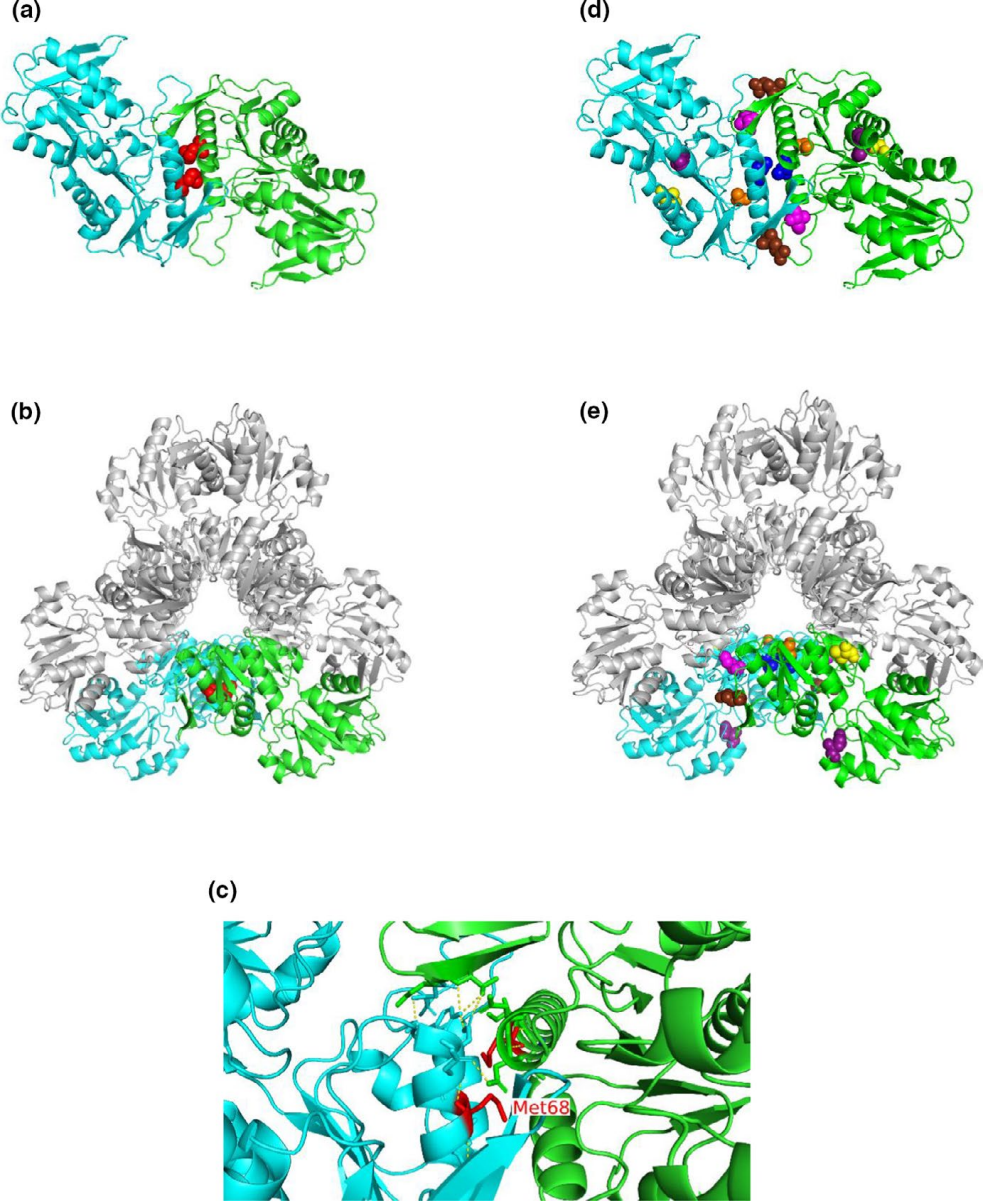
PRPS1 3D model and CMTX5 variants location. Amino acid involved in CMT5X are presented in red balls: Met68 (red) on the dimerization interface for (a–c); Glu43 (brown), Ile107 (pink), Met115 (blue), Ala121 (orange), Gln277 (purple), and Val309 (yellow) for (d and e). PRPS1 dimer structure is represented with one monomer in blue and the other one in green. In the hexamer structure, one dimer is colored and the two other ones are in grey. (a and d) Dimer structure; (b and e) PRPS1 hexamer structure, front view; (c) Focus on the PRPS1 dimerization area and on Met 68

NGS revealed the detection of the hemizygous variant on chromosomic position X:106,882,604, c.202A>T, p.(Met68Leu) (Figure 1). This missense variant was not found in ExAC, dbSNP, Clin Var, and HGMD databases. Neither had it been reported in our French cohort of 3,412 patients suffering from inherited peripheral neuropathies (personal data). Familial segregation was also in accordance with an asymptomatic carrier mother. Therefore, this variant is located in exon 2 corresponding to the N‐terminal domain of the protein. This variant is located in a crucial region, the flag region of the dimer interface (Figure 2). This change is predicted to lead to an altered affinity between the different subunits in the dimer, thereby may prevent the hexamer formation. This substitution may result in the total absence of the PRPS1 hexamer. Therefore in Figure 2, one can see that Met 68 (in red), is located in the alpha helix structure and that the direct surrounding amino acids are involved in dimer interactions. We identified no other potential pathogenic variants in any other screened genes and analysis of the DFNB1 locus also did not reveal any pathogenic variants.

## 4 DISCUSSION

We report the case of a 35‐year‐old male who had developed severe mixed sensory motor polyneuropathy since the age of eight associated to profound deafness. This patient had a new missense variant, c.202A>T, p.(Met68Leu), located on the head of the protein, in the dimerization region.

Phosphoribosyl pyrophosphate synthetase 1 (PRSP1) is an essential enzyme in the primary stage of de novo and salvage nucleotide synthesis, as it catalyzes the phosphoribosylation of ribose 5‐phosphate and ATP to 5‐phosphoribosyl‐1‐pyrophosphate (Becker, 1990). It acts as a common substrate for a purine (adenine and guanine), pyrimidine (cytosine and thymine), and pyridine nucleotide (NAD and NADP) synthesis. The functional form of the enzyme has been shown to have a hexameric structure. The PRPS1 monomer has five‐stranded parallel β sheets and four α‐helices on each of the N‐ and C‐terminal domains, flanked by a short antiparallel β sheet protruding from the central core (a “flag” region). In addition to catalytic and regulatory binding sites, PRPS1 has functional residues involved in inter‐subunit interactions and in maintaining the stability of the enzyme (Kim, 2007). The physiologically active PRS unit is a hexamer that consists of three homodimers arranged in a propeller‐like shape, each with an active site and two regulatory allosteric sites, I and II. The active site comprises binding sites for both ATP and ribose‐5‐phosphate and is located at the interface of two domains within one homodimer (de Brouwer, 2010). Three PRPS genes have been identified; PRPS1 and PRPS2 are widely expressed, while PRPS3 is transcribed only in testis.

The *PRPS1* gene contains seven exons. Missense variants in *PRPS1* are rare and may result in increased or decreased PRS‐I activity (Kim, 2007; Nishikura, 2018; Park, 2013; Robusto, 2015; Synofzik, 2014). The missense variants in CMTX5 are located in exons 2 and 3, close to the flag region/dimerization domain and/or ATP binding site, except for the Synofzik et al patient in exon 6 (Synofzik, 2014). In our case, the variant is located at the center of the dimerization zone, between two homopolymers. We hypothesize that change will probably induce an absence of dimerization, and then an absence of hexamer creation. Five of the other CMT5X variants, p.(Glu43Asp), p.(Ile107Val), p.(Met115Val), p(Met115Thr) and potentially p.(Ala121Gly) are located in or close to this dimerization area. The other two variants, p.(Gln277Pro) and p.(Val309Phe) are located at different sites, and do not seem to be linked to a dimerization problem.

When we look closer to the phenotypes of the patients who harbor pathogenic variants in proximity to the dimer interface (Kim, 2007; Nishikura, 2018; Robusto, 2015) and to our patient's phenotype, a quiet homogeneity comes out. Indeed, all young men patients came from Europe or Asia, presented mixed polyneuropathy started before 10 years of age, prelingual hearing loss and early‐onset optic neuropathy. Only missense variants were found. The specificity of our patient was a more severe damage of the lower limbs and bilateral optic neuropathy was reported.

In a series of cases from one German family harboring p.(Gln277Pro), enzyme activity had been tested and was not detectable in the male index patient, was reduced in the symptomatic carrier sister and normal in the asymptomatic carrier mother (Synofzik, 2014). Thus, a correlation is suggested between the enzymatic residual activity, the degree of X chromosome inactivation skewing and the phenotype in the female. As a consequence, the location of the variant and the residual enzymatic activity could be the main determinants of the phenotypic manifestations in males and females (Synofzik, 2014).

The spectrum of recognized disorders caused by disrupted PRS‐1 function is broad and ranges from adult‐onset gout to severe neurological impairment in childhood. Thus, the variants in the *PRPS1* gene can lead to different conditions due to hypoactivity, such as X‐linked CMT type 5 (CMTX5), Arts syndrome and X‐linked deafness (DFNX1); or PRS superactivity, which can induce gout (Agrahari, 2018).

The CMTX5 disease is characterized by a unique symptom triad of peripheral neuropathy, optic neuropathy, and early‐onset (prelingual) bilateral profound sensorineural hearing loss. Homogeneity is observed between the different patients, especially for age at onset and severity. Mixed sensory motor polyneuropathy before the age of 10, prelingual severe to profound sensorineural hearing loss, optic atrophy during teen ages (Table 1). Electrophysiology has revealed mixed features of segmental demyelination and axonal loss (Kim, 2007). In our patient, sural nerve biopsy did not show any sign of demyelination in childhood. It would have been interesting to repeat the study of the sural nerve biopsy in adulthood, so as to look for any new sign of demyelination. Neither mental retardation nor recurrent infections have been observed.

**Table 1:** Phenotypes and genotypes of our patient and those from the literature. (M: male, NA: not available/:no data, R5P: Ribose5Phosphate)

				Polyneuropathy		Hearing loss		Optic neuropathy		Other symptoms	Mutation type	Zygosity	Nucleotide change	Amino‐acid change	Localisation	Protein domain
Reference	Family	Patient (gender/age in years)	Country	Neuropathy	Age at onset (years)	Degree	Age at onset (years)	Degree	Age at onset (years)							
Kim et al. (2007)	I	M (3‐32)	Europe	Mixed polyneuropathy	< 5	NA	<3	NA	20	None	Missense	Hemizygous heterozygous	c.129A>C	p.(Glu43Asp)	Exon 2	Flag region close to the dimerization domain
	II	M (4‐28)	Korea	Mixed polyneuropathy	10	NA	<3	NA	10	None	Missense	Hemizygous heterozygous	c.344T>C	p.(Met115Thr)	Exon 3	Dimerization domain
Park et al. (2013)	III	M, 15	Korea	Sensory motor mixed polyneuropathy, more severe in the lower limbs	6	Severe to Profound	<1	Absent		None	Missense	Hemizygous heterozygous	c.362C>G	p.(Ala121Gly)	Exon 3	ATP binding site
Synofzik et al. (2014)	IV	M, 36	Germany	Mixed polyneuropathy	30	Severe to Profound	<1	NA	12	Recurrent severe infections, Bulbar paresis Flaccid tetraparesis Ataxia Behavioural and mental disturbances	Missense	Hemizygous heterozygous	c.830A>C	p.(Gln277Pro)	Exon 6	C‐terminal domain, ATP and R5P binding sites
Robusto et al. (2015)	V	M, 12	Italy	Axonal sensory motor neuropathy	<12	Moderate to Profound Progressive	<10	Absent		None	Missense	Hemizygous heterozygous	c.343A>G	p.(Met115Val)	Exon 3	Dimerization domain
	VI	M, 14		Axonal motor neuropathy	<14	Moderate to Profound Progressive	<10				Missense	Hemizygous heterozygous	c.925G>T	p.(Val309Phe)	Exon 7	Type 1 PRTases domain
Nishikura et al. (2018)	VII	M (13‐NA)	Japan	Sensory motor mixed polyneuropathy	8	NA	<1	Absent		Transient proximal muscle weakness, Gowers’ sign and waddling gait after febrile illness	Missense	Hemizygous heterozygous	c.319A>G	p.(Ile107Val)	Exon 3	Dimerization domain
Our study	VIII	M, 35	France	Sensory motor mixed polyneuropathy, more severe in the lower limbs	8	Profound	<1	Bilateral	NA	None	Missense	Hemizygous heterozygous	**c.202A>T**	**p.(Met68Leu)**	Exon 2	Dimerization domain

Synofzik *et al* described a male with an intermediate phenotype between CMTX5 and Arts syndrome and a carrier female affected with DFN2 due to X‐chromosome inactivation skewing (Synofzik, 2014). Nevertheless, phenotypic heterogeneity exists among patients with CMTX5 which explains why a Korean family has been reported with CMTX5 features without any sign of optic atrophy (Park, 2013). The burden between the different forms of diseases due to *PRPS1* pathogenic variants is very narrow, and some conditions are at the cross lines between two conditions. The severity and progression of CMTX5 phenotypes vary according to the sites of the *PRPS1* pathogenic variants (Park, 2013). An overlap of CMTX5 and Arts syndrome has been reported with the pathogenic variant c.830A>C, p.(Gln277Pro), and also X‐linked hearing loss and neuropathy with pathogenic variants c.337G>T, p.(Ala113Ser) and c.925G>T p.(Val309Phe) (Robusto, 2015; Synofzik, 2014). For the overlap between CMTX5 and Arts syndrome, cerebral MRI has revealed cerebellar and parietal atrophy, evidence of structural central nervous system damage (Synofzik, 2014, 2014). Nishikura et al have also recently reported this overlap between CMTX5 and Arts syndrome with transient proximal weakness, showing Gower's sign and waddling gait after suffering from febrile illness (Nishikura, 2018).

Among a same family, symptoms can also differ. Female carriers might exhibit mild symptoms in case of CMTX5 (Kim, 2007; Robusto, 2015). Affected females have been described by Almoguera et al in a three‐generation Spanish family, with optic atrophy and retinitis pigmentosa as common female features (Almoguera, 2014). Ataxia, progressive peripheral neuropathy and hearing loss could be possible variable additional features. This was due to the pathogenic variant c.46T>C, p.(Ser16Pro). Fiorentino et al have recently shown that affected females presenting retinal dystrophy with interocular asymmetry in five families with nine affected women due to pathogenic variants c.47C>T, p.(Ser16Phe); c.586C>T, p.(Arg196Trp); c.641G>C, p.(Arg214Pro), and c.640C>T, p.(Arg214Trp). Sensorineural hearing loss was reported in three cases, it was progressive and had started during childhood but had been delayed. It was linked to pathogenic variants c.641G>C, p.(Arg214Pro), and c.640C>T, p.(Arg214Trp). Thus, one differential diagnosis of this presentation is Usher syndrome (Fiorentino, 2018). As only females were affected in this cohort of five families, we may think that, when inherited in the hemizygous state in males, this could be male embryonic lethal (Fiorentino, 2018).

The phenotypic presentation in males is determined by the exact *PRPS1* pathogenic variant, its structural effect on the enzyme and the residual enzyme activity. There is no way to completely rule out the possibility that other pathogenic variants in coding or regulatory regions of the genome may contribute to modifying the phenotype.

For loss‐of‐function pathogenic variants resulting in sensorineural hearing loss, Sadenosylmethionine (SAM) supplements in patients with Arts syndrome help to replenish ATP and GTP concentrations to some extent, thereby slowing the progression of sensorineural hearing loss and alleviating some of the neurological symptoms (Mittal et al., 2015).

## 5 CONCLUSION

CMTX5 is a rare condition, with only seven pathogenic variants already reported. However, it is presumably under‐diagnosed, as an overlap among the different features due to *PRPS1* exists. Patients who developed polyneuropathy associated to sensorineural deafness and optic atrophy during childhood should be assessed for *PRPS1.* Here, we report the first novel pathogenic variant in the French population. Moreover, it is located at the center of the dimerization zone, between two homopolymers. We hypothesize that change may induce an absence of dimerization, and then an absence of hexamer creation.

## CONFLICT OF INTEREST

The authors declare no conflict of interest.
