# Supplementary material for: New PRPS1 variant p.(Met68Leu) located in the dimerization area identified in a French CMTX5 patient
Source: Mol Genet Genomic Med. 2019 Jul 23;7(9):e875. doi: 10.1002/mgg3.875 (PMC6732271; doi:10.1002/mgg3.875)
Supplement: Supplementary file 1 [file MGG3-7-e875-s001.docx]

**SUPPLEMENTARY DATA**

*Supp Table 1: 92-gene panel used for NGS. It includes the 44 known CMT genes, 27 genes involved in HSN (Hereditary Sensitive Neuropathy) and HMN (Hereditary Motor Neuropathy) and 21 other genes of interest involved in neuropathies of differential diagnoses.*

| GENE | CMT1D | CMT2D | CMT1R | CMT2R | HMN D | HMNR | HSN D | HSN R | Other |
| --- | --- | --- | --- | --- | --- | --- | --- | --- | --- |
| *AARS* |  | X |  |  |  |  |  |  |  |
| *ABHD12* |  |  |  |  |  |  |  |  | R |
| *AIFM1* |  |  |  | X |  |  |  |  |  |
| *ARHGEF10* |  |  |  |  |  |  |  |  | D |
| *ATL1* |  |  |  |  |  |  | X |  |  |
| *ATL3* |  |  |  |  |  |  | X |  |  |
| *ATP7A* |  |  |  |  |  | X |  |  |  |
| *BICD2* |  |  |  |  | X |  |  |  |  |
| *BSCL2* |  |  |  |  | X |  |  |  |  |
| *CCT5* |  |  |  |  |  |  |  | X |  |
| *CTDP1* |  |  |  |  |  |  |  | X |  |
| *C12ORF65* |  |  |  |  |  |  |  |  | R |
| *DCAF8* |  |  |  |  |  |  |  |  | D |
| *DCTN1* |  |  |  |  | X |  |  |  |  |
| *DHTKD1* |  | X |  |  |  |  |  |  |  |
| *DNAJB2* |  |  |  | X |  |  |  |  |  |
| *DNM2* | X | X |  |  |  |  |  |  |  |
| *DNMT1* |  |  |  |  |  |  | X |  |  |
| *DST* |  |  |  |  |  |  |  | X |  |
| *DYNC1H1* |  | X |  |  |  |  |  |  |  |
| *EGR2* | X |  | X |  |  |  |  |  |  |
| *FAM134B* |  |  |  |  |  |  |  | X |  |
| *FBLN5* |  |  |  |  |  |  |  |  | D/R |
| *FBXO38* |  |  |  |  | X |  |  |  |  |
| *FGD4* |  |  | X |  |  |  |  |  |  |
| *FIG4* |  |  | X |  | X |  |  |  |  |
| *GAN* |  |  |  |  |  |  |  |  | R |
| *GARS* |  | X |  |  | X |  |  |  |  |
| *GDAP1* |  | X | X | X |  |  |  |  |  |
| *GJB1* | X | X |  |  |  |  |  |  |  |
| *GJB3* |  |  |  |  |  |  |  |  | D/R |
| *GNB4* | X | X |  |  |  |  |  |  |  |
| *HARS* |  | X |  |  |  |  |  |  |  |
| *HINT1* |  |  |  |  |  |  |  |  | R |
| *HK1* |  |  | X |  |  |  |  |  |  |
| *HSPB1* |  | X |  |  | X |  |  |  |  |
| *HSPB3* |  |  |  |  | X |  |  |  |  |
| *HSPB8* |  | X |  |  | X |  |  |  |  |
| *IFRD1* |  |  |  |  |  |  |  |  | X |
| *IGHMBP2* |  |  |  | X |  | X |  |  |  |
| *IKBKAP* |  |  |  |  |  |  |  | X |  |
| *INF2* | X | X |  |  |  |  |  |  |  |
| *KARS* |  |  | X | X |  |  |  |  |  |
| *KIF1A* |  |  |  |  |  |  |  | X |  |
| *KIF1B* |  | X |  |  |  |  |  |  |  |
| *KIF5A* |  |  |  |  |  |  |  |  | D |
| *LITAF* | X |  |  |  |  |  |  |  |  |
| *LMNA* |  |  |  | X |  |  |  |  |  |
| *LRSAM1* |  | X |  |  |  |  |  |  |  |
| *MARS* |  | X |  |  |  |  |  |  |  |
| *MED25* |  |  |  | X |  |  |  |  |  |
| *MFN2* |  | X |  |  |  |  |  |  |  |
| *MPV17* |  |  |  |  |  |  |  |  | R |
| *MPZ* | X | X | X |  |  |  |  |  |  |
| *MTMR2* |  |  | X |  |  |  |  |  |  |
| *NDRG1* |  |  | X |  |  |  |  |  |  |
| *NEFL* | X | X |  |  |  |  |  |  |  |
| *NGF* |  |  |  |  |  |  |  | X |  |
| *NTRK1* |  |  |  |  |  |  |  | X |  |
| *PDK3* |  | X |  |  |  |  |  |  |  |
| *PLEKHG5* |  |  | X | X |  | X |  |  |  |
| *PMP22* | X |  |  |  |  |  |  |  |  |
| *POLG* |  |  |  |  |  |  |  |  | D/R |
| *PRPS1* |  |  |  | X |  |  |  |  |  |
| *PRX* |  |  | X |  |  |  |  |  |  |
| *RAB7A* |  | X |  |  |  |  |  |  |  |
| *REEP1* |  |  |  |  | X |  |  |  |  |
| *SBF1* |  |  | X |  |  |  |  |  |  |
| *SBF2* |  |  | X |  |  |  |  |  |  |
| *SCN9A* |  |  |  |  |  |  |  | X |  |
| *SCN10A* |  |  |  |  |  |  | X |  |  |
| *SCN11A* |  |  |  |  |  |  | X |  |  |
| *SEPT9* |  |  |  |  |  |  |  |  | *D* |
| *SETX* |  |  |  |  | X |  |  |  |  |
| *SH3TC2* |  |  | X |  |  |  |  |  |  |
| *SLC12A6* |  |  |  |  |  |  |  |  | R |
| *SLC5A7* |  |  |  |  | X |  |  |  |  |
| *SMAD3* |  |  |  |  |  |  |  |  | D |
| *SOX10* |  |  |  |  |  |  |  |  | D |
| *SPTLC1* |  |  |  |  |  |  | X |  |  |
| *SPTLC2* |  |  |  |  |  |  | X |  |  |
| *SURF1* |  |  | X |  |  |  |  |  |  |
| *TFG* |  |  |  |  |  |  |  |  | D |
| *TRIM2* |  |  |  | X |  |  |  |  |  |
| *TRPV4* |  | X |  |  | X |  |  |  |  |
| *TTR* |  |  |  |  |  |  |  |  | D |
| *TUBB3* |  |  |  |  |  |  |  |  | D |
| *UBQLN2* |  |  |  |  | X |  |  |  |  |
| *VAPB* |  |  |  |  |  |  |  |  | D |
| *VCP* |  |  |  |  |  |  |  |  | D |
| *WNK1* |  |  |  |  |  |  |  | X |  |
| *YARS* | X | X |  |  |  |  |  |  |  |
